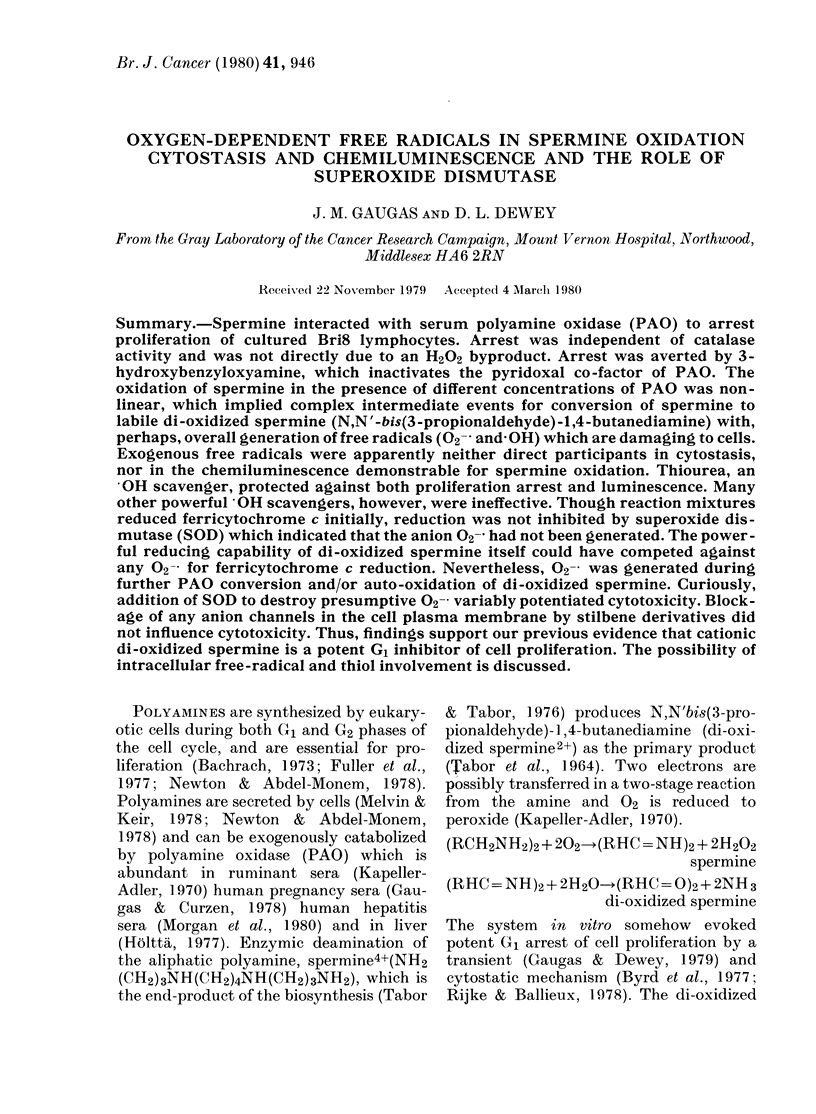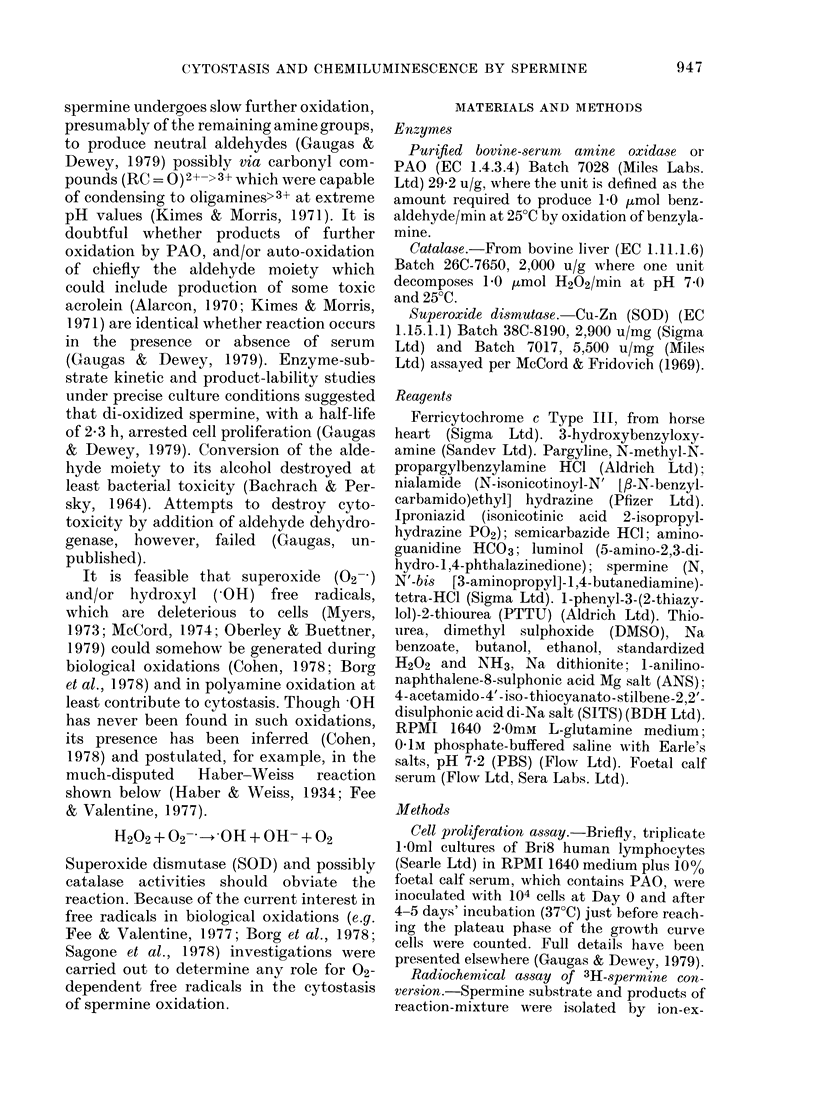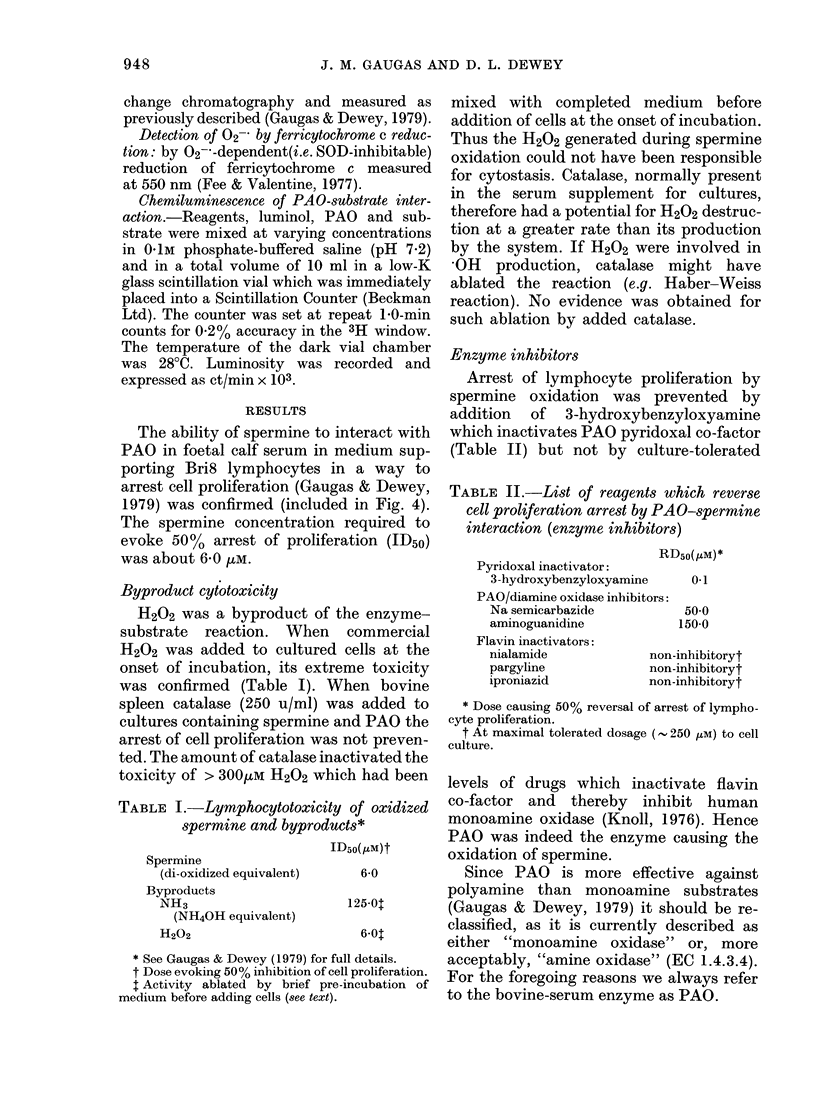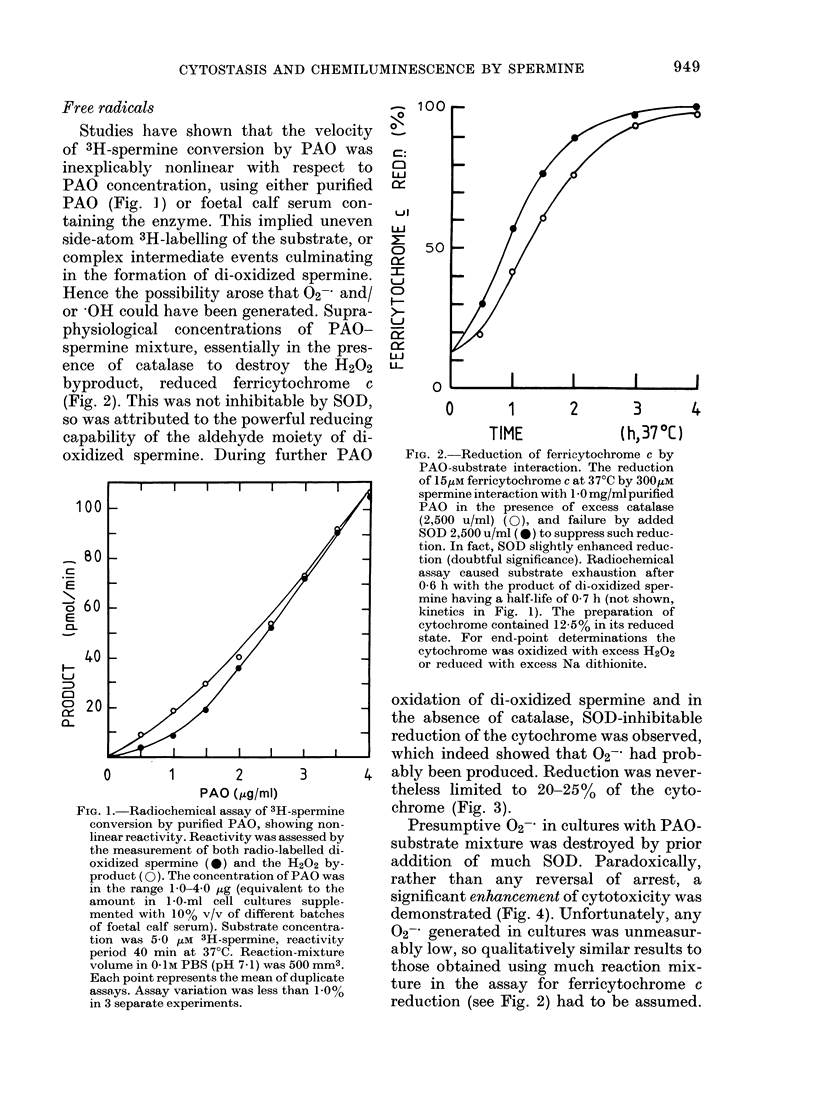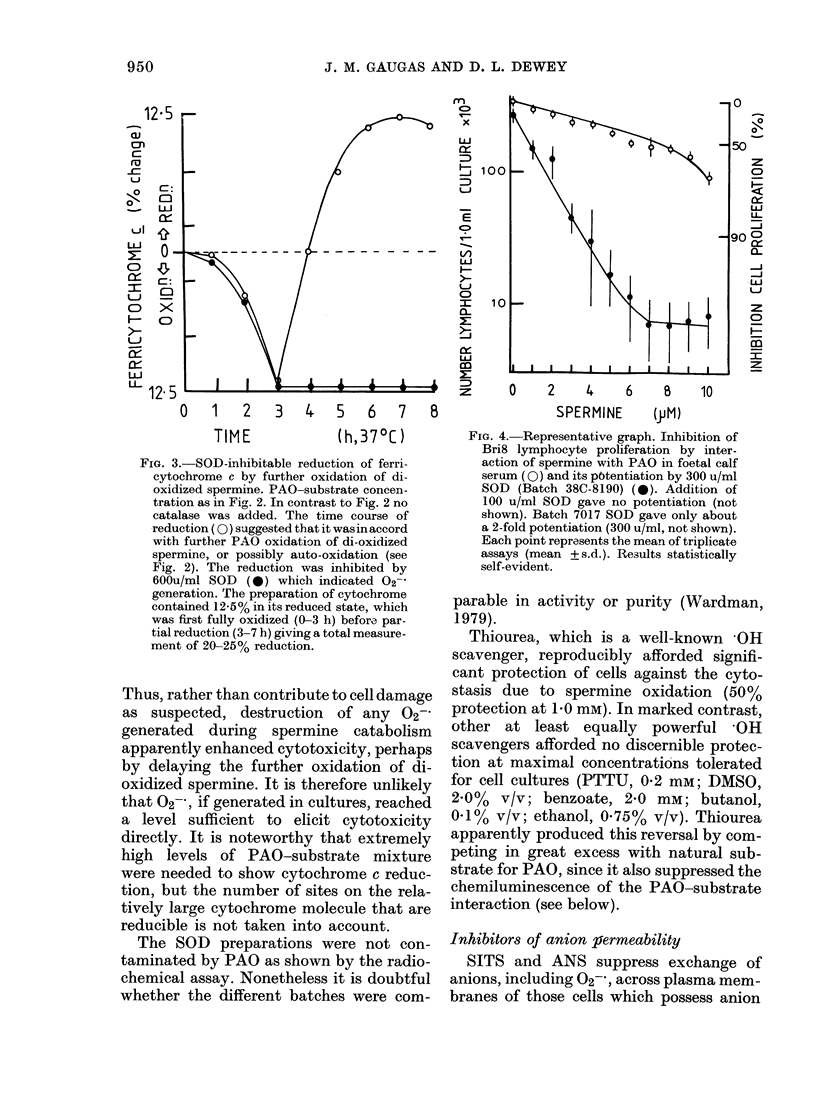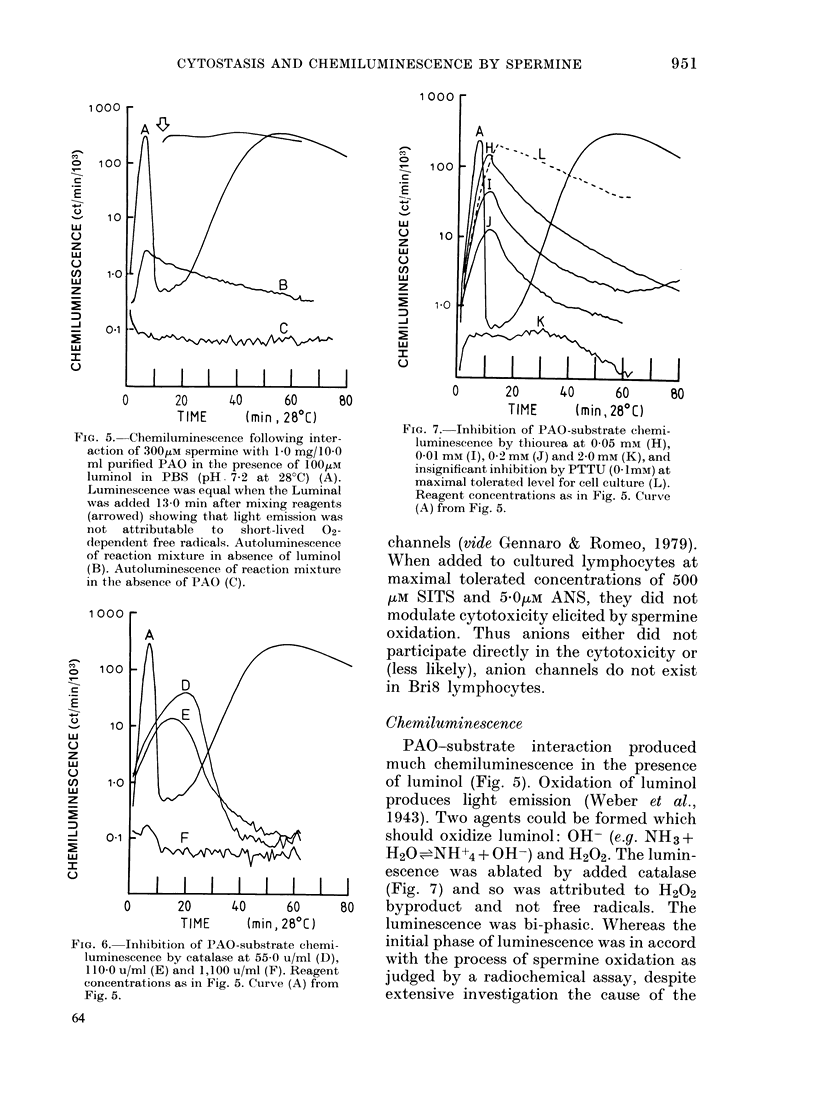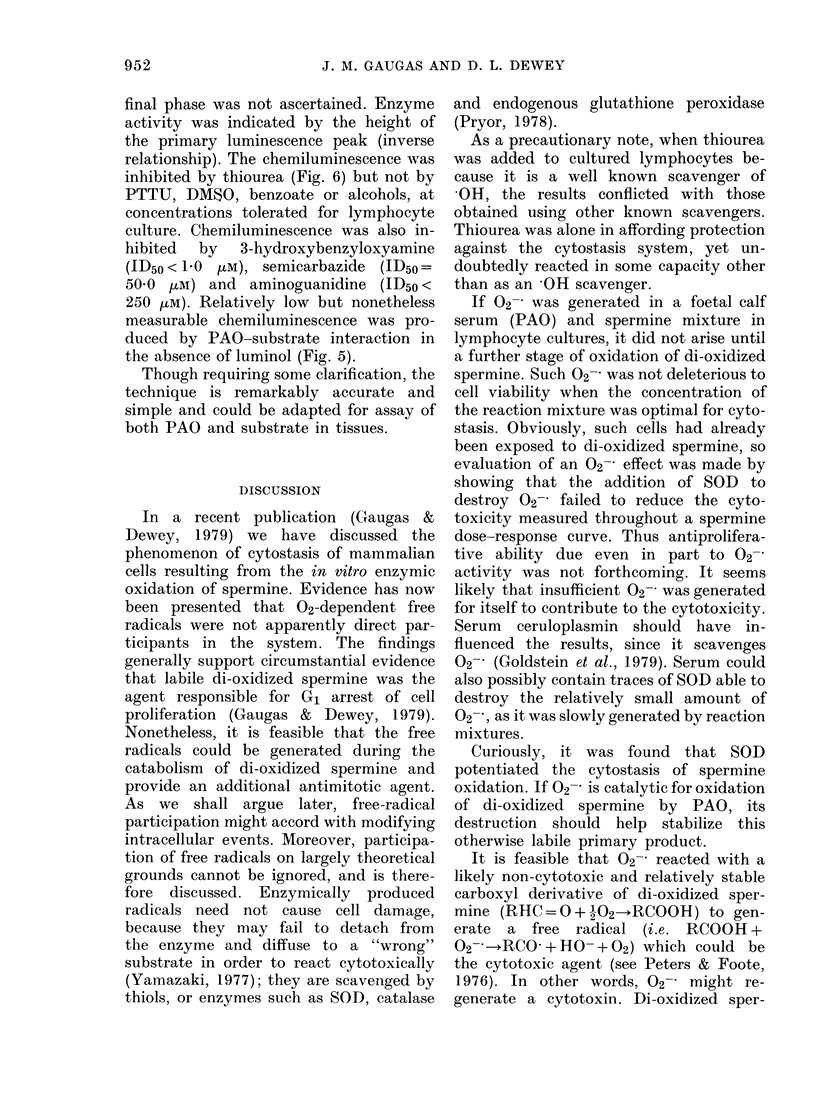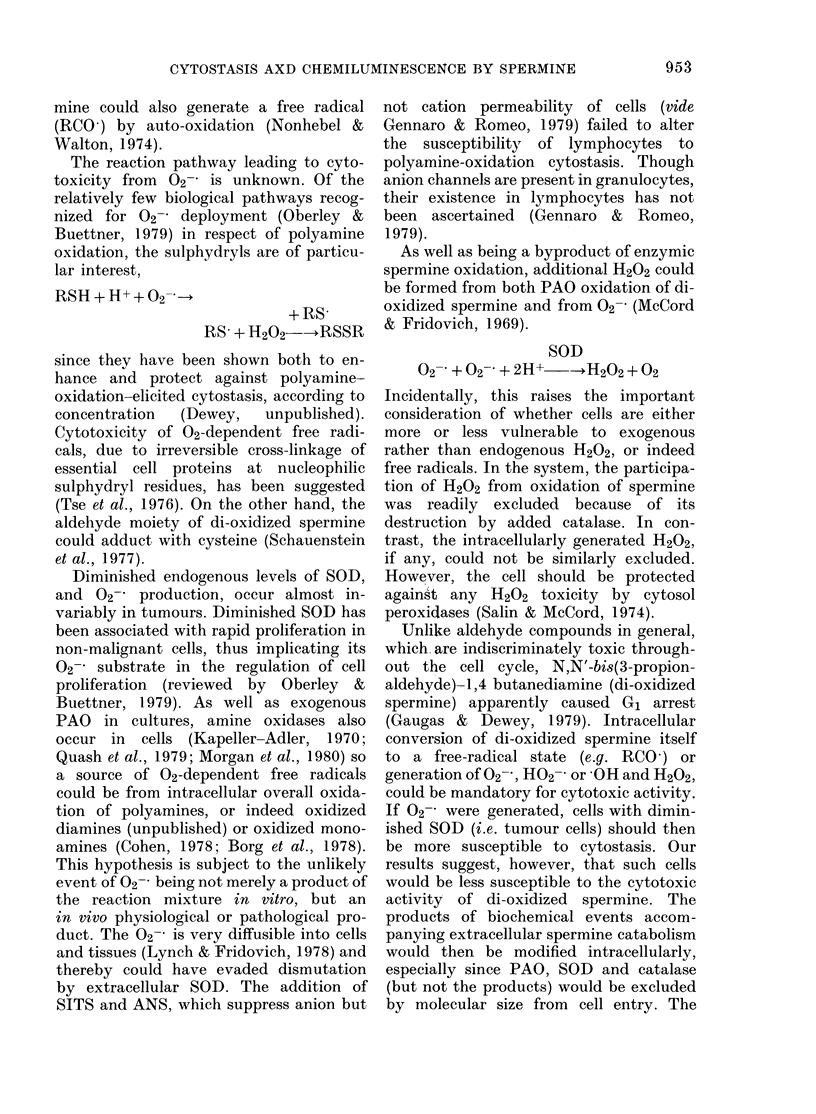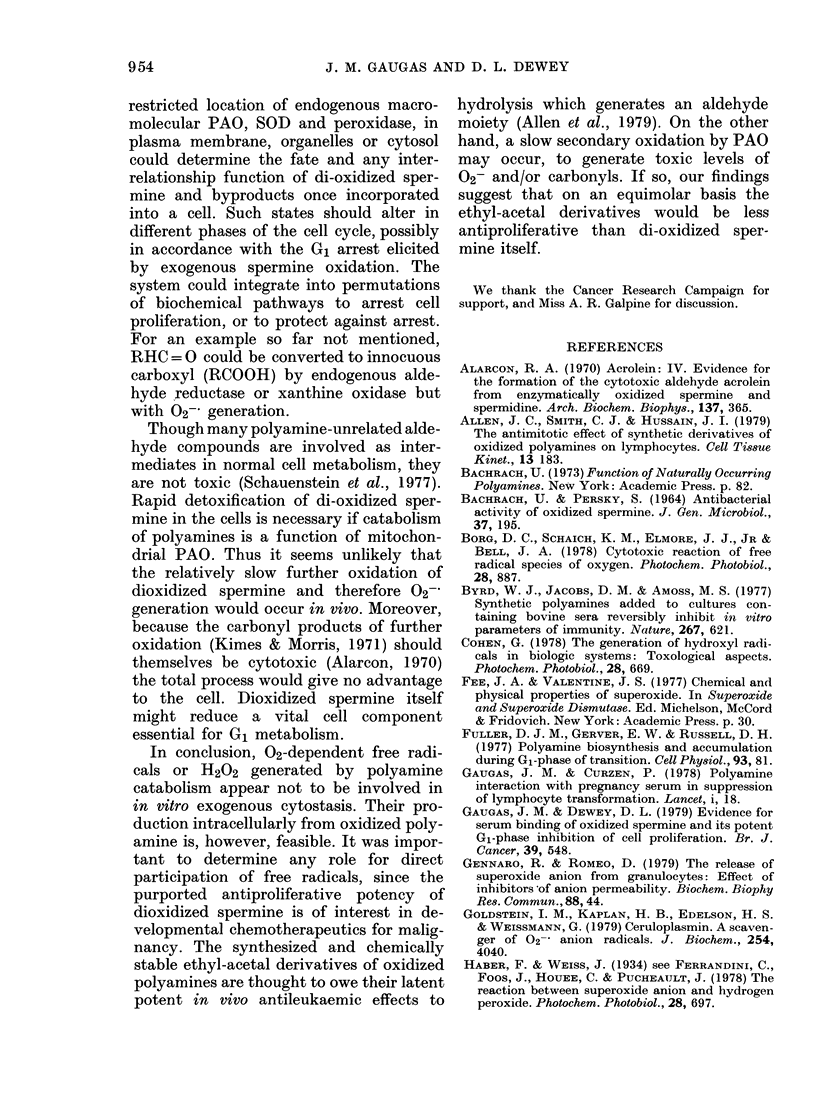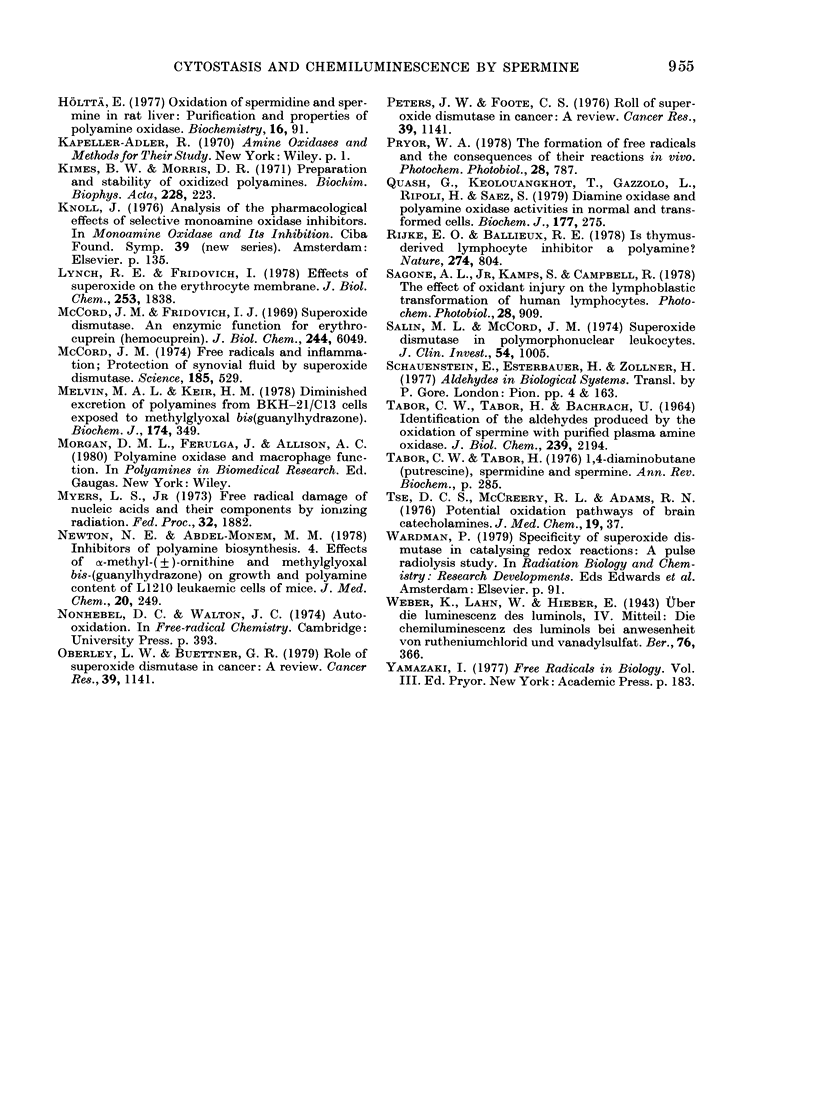# Oxygen-dependent free radicals in spermine oxidation cytostasis and chemiluminescence and the role of superoxide dismutase

**DOI:** 10.1038/bjc.1980.173

**Published:** 1980-06

**Authors:** J. M. Gaugas, D. L. Dewey

## Abstract

Spermine interacted with serum polyamine oxidase (PAO) to arrest proliferation of cultured Bri8 lymphocytes. Arrest was independent of catalase activity and was not directly due to an H_2_O_2_ byproduct. Arrest was averted by 3-hydroxybenzyloxyamine, which inactivates the pyridoxal co-factor of PAO. The oxidation of spermine in the presence of different concentrations of PAO was non-linear, which implied complex intermediate events for conversion of spermine to labile di-oxidized spermine (N,N′-*bis*(3-propionaldehyde)-1,4-butanediamine) with, perhaps, overall generation of free radicals (O_2_^-·^ and ·OH) which are damaging to cells. Exogenous free radicals were apparently neither direct participants in cytostasis, nor in the chemiluminescence demonstrable for spermine oxidation. Thiourea, an ·OH scavenger, protected against both proliferation arrest and luminescence. Many other powerful ·OH scavengers, however, were ineffective. Though reaction mixtures reduced ferricytochrome *c* initially, reduction was not inhibited by superoxide dismutase (SOD) which indicated that the anion O_2_^-·^ had not been generated. The powerful reducing capability of di-oxidized spermine itself could have competed against any O_2_^-·^ for ferricytochrome *c* reduction. Nevertheless, O_2_^-·^ was generated during further PAO conversion and/or auto-oxidation of di-oxidized spermine. Curiously, addition of SOD to destroy presumptive O_2_^-·^ variably potentiated cytotoxicity. Blockage of any anion channels in the cell plasma membrane by stilbene derivatives did not influence cytotoxicity. Thus, findings support our previous evidence that cationic di-oxidized spermine is a potent G_1_ inhibitor of cell proliferation. The possibility of intracellular free-radical and thiol involvement is discussed.


*Br. J. Cancer* (1980) **41**, 946

### OXYGEN-DEPENDENT FREE RADICALS IN SPERMINE OXIDATION CYTOSTASIS AND CHEMILUMINESCENCE AND THE ROLE OF SUPEROXIDE DISMUTASE

J. M. GAUGAS AND D. L. DEWEY

*From the Gray Laboratory of the Cancer Research Campaign, Mount Vernon Hospital, Northwood, Middlesex HA6 2RN*

Received 22 November 1979 Accepted 4 March 1980

**Summary.—Spermine interacted with serum polyamine oxidase (PAO) to arrest proliferation of cultured Bri8 lymphocytes. Arrest was independent of catalase activity and was not directly due to an $H_2O_2$ byproduct. Arrest was averted by 3-hydroxybenzyloxyamine, which inactivates the pyridoxal co-factor of PAO. The oxidation of spermine in the presence of different concentrations of PAO was non-linear, which implied complex intermediate events for conversion of spermine to labile di-oxidized spermine (N,N′-*bis*(3-propionaldehyde)-1,4-butanediamine) with, perhaps, overall generation of free radicals ($O_2^{-\cdot}$ and $\cdot OH$) which are damaging to cells. Exogenous free radicals were apparently neither direct participants in cytostasis, nor in the chemiluminescence demonstrable for spermine oxidation. Thiourea, an $\cdot OH$ scavenger, protected against both proliferation arrest and luminescence. Many other powerful $\cdot OH$ scavengers, however, were ineffective. Though reaction mixtures reduced ferricytochrome *c* initially, reduction was not inhibited by superoxide dismutase (SOD) which indicated that the anion $O_2^{-\cdot}$ had not been generated. The powerful reducing capability of di-oxidized spermine itself could have competed against any $O_2^{-\cdot}$ for ferricytochrome *c* reduction. Nevertheless, $O_2^{-\cdot}$ was generated during further PAO conversion and/or auto-oxidation of di-oxidized spermine. Curiously, addition of SOD to destroy presumptive $O_2^{-\cdot}$ variably potentiated cytotoxicity. Blockage of any anion channels in the cell plasma membrane by stilbene derivatives did not influence cytotoxicity. Thus, findings support our previous evidence that cationic di-oxidized spermine is a potent $G_1$ inhibitor of cell proliferation. The possibility of intracellular free-radical and thiol involvement is discussed.**

POLYAMINES are synthesized by eukaryotic cells during both $G_1$ and $G_2$ phases of the cell cycle, and are essential for proliferation (Bachrach, 1973; Fuller *et al.*, 1977; Newton & Abdel-Monem, 1978). Polyamines are secreted by cells (Melvin & Keir, 1978; Newton & Abdel-Monem, 1978) and can be exogenously catabolized by polyamine oxidase (PAO) which is abundant in ruminant sera (Kapeller-Adler, 1970) human pregnancy sera (Gaugas & Curzen, 1978) human hepatitis sera (Morgan *et al.*, 1980) and in liver (Höltta, 1977). Enzymic deamination of the aliphatic polyamine, spermine$^{4+}$($NH_2(CH_2)_3NH(CH_2)_4NH(CH_2)_3NH_2$), which is the end-product of the biosynthesis (Tabor & Tabor, 1976) produces N,N′*bis*(3-propionaldehyde)-1,4-butanediamine (di-oxidized spermine$^{2+}$) as the primary product (Tabor *et al.*, 1964). Two electrons are possibly transferred in a two-stage reaction from the amine and $O_2$ is reduced to peroxide (Kapeller-Adler, 1970).

$$\underset{\text{spermine}}{(RCH_2NH_2)_2 + 2O_2 \rightarrow (RHC{=}NH)_2 + 2H_2O_2}$$

$$\underset{\text{di-oxidized spermine}}{(RHC{=}NH)_2 + 2H_2O \rightarrow (RHC{=}O)_2 + 2NH_3}$$

The system *in vitro* somehow evoked potent $G_1$ arrest of cell proliferation by a transient (Gaugas & Dewey, 1979) and cytostatic mechanism (Byrd *et al.*, 1977; Rijke & Ballieux, 1978). The di-oxidized spermine undergoes slow further oxidation, presumably of the remaining amine groups, to produce neutral aldehydes (Gaugas & Dewey, 1979) possibly *via* carbonyl compounds $(RC=O)^{2+->3+}$ which were capable of condensing to oligamines$^{>3+}$ at extreme pH values (Kimes & Morris, 1971). It is doubtful whether products of further oxidation by PAO, and/or auto-oxidation of chiefly the aldehyde moiety which could include production of some toxic acrolein (Alarcon, 1970; Kimes & Morris, 1971) are identical whether reaction occurs in the presence or absence of serum (Gaugas & Dewey, 1979). Enzyme-substrate kinetic and product-lability studies under precise culture conditions suggested that di-oxidized spermine, with a half-life of 2·3 h, arrested cell proliferation (Gaugas & Dewey, 1979). Conversion of the aldehyde moiety to its alcohol destroyed at least bacterial toxicity (Bachrach & Persky, 1964). Attempts to destroy cytotoxicity by addition of aldehyde dehydrogenase, however, failed (Gaugas, unpublished).

It is feasible that superoxide ($O_2^{-\cdot}$) and/or hydroxyl ($\cdot OH$) free radicals, which are deleterious to cells (Myers, 1973; McCord, 1974; Oberley & Buettner, 1979) could somehow be generated during biological oxidations (Cohen, 1978; Borg *et al.*, 1978) and in polyamine oxidation at least contribute to cytostasis. Though $\cdot OH$ has never been found in such oxidations, its presence has been inferred (Cohen, 1978) and postulated, for example, in the much-disputed Haber–Weiss reaction shown below (Haber & Weiss, 1934; Fee & Valentine, 1977).

$$H_2O_2 + O_2^{-\cdot} \rightarrow \cdot OH + OH^- + O_2$$

Superoxide dismutase (SOD) and possibly catalase activities should obviate the reaction. Because of the current interest in free radicals in biological oxidations (*e.g.* Fee & Valentine, 1977; Borg *et al.*, 1978; Sagone *et al.*, 1978) investigations were carried out to determine any role for $O_2$-dependent free radicals in the cytostasis of spermine oxidation.

### MATERIALS AND METHODS

### *Enzymes*

*Purified bovine-serum amine oxidase* or PAO (EC 1.4.3.4) Batch 7028 (Miles Labs. Ltd) 29·2 u/g, where the unit is defined as the amount required to produce 1·0 $\mu$mol benzaldehyde/min at 25°C by oxidation of benzylamine.

*Catalase.*—From bovine liver (EC 1.11.1.6) Batch 26C-7650, 2,000 u/g where one unit decomposes 1·0 $\mu$mol $H_2O_2$/min at pH 7·0 and 25°C.

*Superoxide dismutase.*—Cu-Zn (SOD) (EC 1.15.1.1) Batch 38C-8190, 2,900 u/mg (Sigma Ltd) and Batch 7017, 5,500 u/mg (Miles Ltd) assayed per McCord & Fridovich (1969).

### *Reagents*

Ferricytochrome *c* Type III, from horse heart (Sigma Ltd). 3-hydroxybenzyloxyamine (Sandev Ltd). Pargyline, N-methyl-N-propargylbenzylamine HCl (Aldrich Ltd); nialamide (N-isonicotinoyl-N′ [$\beta$-N-benzylcarbamido)ethyl] hydrazine (Pfizer Ltd). Iproniazid (isonicotinic acid 2-isopropylhydrazine $PO_2$); semicarbazide HCl; aminoguanidine $HCO_3$; luminol (5-amino-2,3-dihydro-1,4-phthalazinedione); spermine (N, N′-*bis* [3-aminopropyl]-1,4-butanediamine)-tetra-HCl (Sigma Ltd). 1-phenyl-3-(2-thiazylol)-2-thiourea (PTTU) (Aldrich Ltd). Thiourea, dimethyl sulphoxide (DMSO), Na benzoate, butanol, ethanol, standardized $H_2O_2$ and $NH_3$, Na dithionite; 1-anilinonaphthalene-8-sulphonic acid Mg salt (ANS); 4-acetamido-4′-iso-thiocyanato-stilbene-2,2′-disulphonic acid di-Na salt (SITS) (BDH Ltd). RPMI 1640 2·0mM L-glutamine medium; 0·1M phosphate-buffered saline with Earle's salts, pH 7·2 (PBS) (Flow Ltd). Foetal calf serum (Flow Ltd, Sera Labs. Ltd).

### *Methods*

*Cell proliferation assay.*—Briefly, triplicate 1·0ml cultures of Bri8 human lymphocytes (Searle Ltd) in RPMI 1640 medium plus 10% foetal calf serum, which contains PAO, were inoculated with $10^4$ cells at Day 0 and after 4–5 days' incubation (37°C) just before reaching the plateau phase of the growth curve cells were counted. Full details have been presented elsewhere (Gaugas & Dewey, 1979).

*Radiochemical assay of* $^3$H-*spermine conversion.*—Spermine substrate and products of reaction-mixture were isolated by ion-exchange chromatography and measured as previously described (Gaugas & Dewey, 1979).

*Detection of* $O_2^{-\cdot}$ *by ferricytochrome* c *reduction:* by $O_2^{-\cdot}$-dependent(*i.e.* SOD-inhibitable) reduction of ferricytochrome *c* measured at 550 nm (Fee & Valentine, 1977).

*Chemiluminescence of PAO-substrate interaction.*—Reagents, luminol, PAO and substrate were mixed at varying concentrations in 0·1M phosphate-buffered saline (pH 7·2) and in a total volume of 10 ml in a low-K glass scintillation vial which was immediately placed into a Scintillation Counter (Beckman Ltd). The counter was set at repeat 1·0-min counts for 0·2% accuracy in the $^3H$ window. The temperature of the dark vial chamber was 28°C. Luminosity was recorded and expressed as ct/min × $10^3$.

### RESULTS

The ability of spermine to interact with PAO in foetal calf serum in medium supporting Bri8 lymphocytes in a way to arrest cell proliferation (Gaugas & Dewey, 1979) was confirmed (included in Fig. 4). The spermine concentration required to evoke 50% arrest of proliferation ($ID_{50}$) was about 6·0 $\mu$M.

### Byproduct cytotoxicity

$H_2O_2$ was a byproduct of the enzyme–substrate reaction. When commercial $H_2O_2$ was added to cultured cells at the onset of incubation, its extreme toxicity was confirmed (Table I). When bovine spleen catalase (250 u/ml) was added to cultures containing spermine and PAO the arrest of cell proliferation was not prevented. The amount of catalase inactivated the toxicity of > 300$\mu$M $H_2O_2$ which had been mixed with completed medium before addition of cells at the onset of incubation. Thus the $H_2O_2$ generated during spermine oxidation could not have been responsible for cytostasis. Catalase, normally present in the serum supplement for cultures, therefore had a potential for $H_2O_2$ destruction at a greater rate than its production by the system. If $H_2O_2$ were involved in ·OH production, catalase might have ablated the reaction (*e.g.* Haber–Weiss reaction). No evidence was obtained for such ablation by added catalase.

TABLE I.—*Lymphocytotoxicity of oxidized spermine and byproducts**

| | $ID_{50}$($\mu$M)† |
|---|---|
| Spermine | |
| (di-oxidized equivalent) | 6·0 |
| Byproducts | |
| $NH_3$ ($NH_4OH$ equivalent) | 125·0‡ |
| $H_2O_2$ | 6·0‡ |

* See Gaugas & Dewey (1979) for full details.
† Dose evoking 50% inhibition of cell proliferation.
‡ Activity ablated by brief pre-incubation of medium before adding cells (*see text*).

### Enzyme inhibitors

Arrest of lymphocyte proliferation by spermine oxidation was prevented by addition of 3-hydroxybenzyloxyamine which inactivates PAO pyridoxal co-factor (Table II) but not by culture-tolerated levels of drugs which inactivate flavin co-factor and thereby inhibit human monoamine oxidase (Knoll, 1976). Hence PAO was indeed the enzyme causing the oxidation of spermine.

TABLE II.—*List of reagents which reverse cell proliferation arrest by PAO–spermine interaction (enzyme inhibitors)*

| | $RD_{50}$($\mu$M)* |
|---|---|
| Pyridoxal inactivator: | |
| 3-hydroxybenzyloxyamine | 0·1 |
| PAO/diamine oxidase inhibitors: | |
| Na semicarbazide | 50·0 |
| aminoguanidine | 150·0 |
| Flavin inactivators: | |
| nialamide | non-inhibitory† |
| pargyline | non-inhibitory† |
| iproniazid | non-inhibitory† |

* Dose causing 50% reversal of arrest of lymphocyte proliferation.
† At maximal tolerated dosage (~250 $\mu$M) to cell culture.

Since PAO is more effective against polyamine than monoamine substrates (Gaugas & Dewey, 1979) it should be reclassified, as it is currently described as either "monoamine oxidase" or, more acceptably, "amine oxidase" (EC 1.4.3.4). For the foregoing reasons we always refer to the bovine-serum enzyme as PAO.

*Free radicals*

Studies have shown that the velocity of $^3H$-spermine conversion by PAO was inexplicably nonlinear with respect to PAO concentration, using either purified PAO (Fig. 1) or foetal calf serum containing the enzyme. This implied uneven side-atom $^3H$-labelling of the substrate, or complex intermediate events culminating in the formation of di-oxidized spermine. Hence the possibility arose that $O_2^{-\cdot}$ and/or $\cdot OH$ could have been generated. Supraphysiological concentrations of PAO–spermine mixture, essentially in the presence of catalase to destroy the $H_2O_2$ byproduct, reduced ferricytochrome *c* (Fig. 2). This was not inhibitable by SOD, so was attributed to the powerful reducing capability of the aldehyde moiety of di-oxidized spermine. During further PAO oxidation of di-oxidized spermine and in the absence of catalase, SOD-inhibitable reduction of the cytochrome was observed, which indeed showed that $O_2^{-\cdot}$ had probably been produced. Reduction was nevertheless limited to 20–25% of the cytochrome (Fig. 3).

FIG. 1.—Radiochemical assay of $^3H$-spermine conversion by purified PAO, showing non-linear reactivity. Reactivity was assessed by the measurement of both radio-labelled di-oxidized spermine (●) and the $H_2O_2$ by-product (○). The concentration of PAO was in the range 1·0–4·0 μg (equivalent to the amount in 1·0-ml cell cultures supplemented with 10% v/v of different batches of foetal calf serum). Substrate concentration was 5·0 μM $^3H$-spermine, reactivity period 40 min at 37°C. Reaction-mixture volume in 0·1M PBS (pH 7·1) was 500 mm³. Each point represents the mean of duplicate assays. Assay variation was less than 1·0% in 3 separate experiments.

FIG. 2.—Reduction of ferricytochrome *c* by PAO-substrate interaction. The reduction of 15μM ferricytochrome *c* at 37°C by 300μM spermine interaction with 1·0 mg/ml purified PAO in the presence of excess catalase (2,500 u/ml) (○), and failure by added SOD 2,500 u/ml (●) to suppress such reduction. In fact, SOD slightly enhanced reduction (doubtful significance). Radiochemical assay caused substrate exhaustion after 0·6 h with the product of di-oxidized spermine having a half-life of 0·7 h (not shown, kinetics in Fig. 1). The preparation of cytochrome contained 12·5% in its reduced state. For end-point determinations the cytochrome was oxidized with excess $H_2O_2$ or reduced with excess Na dithionite.

Presumptive $O_2^{-\cdot}$ in cultures with PAO-substrate mixture was destroyed by prior addition of much SOD. Paradoxically, rather than any reversal of arrest, a significant *enhancement* of cytotoxicity was demonstrated (Fig. 4). Unfortunately, any $O_2^{-\cdot}$ generated in cultures was unmeasurably low, so qualitatively similar results to those obtained using much reaction mixture in the assay for ferricytochrome *c* reduction (see Fig. 2) had to be assumed.

FIG. 3.—SOD-inhibitable reduction of ferricytochrome *c* by further oxidation of di-oxidized spermine. PAO–substrate concentration as in Fig. 2. In contrast to Fig. 2 no catalase was added. The time course of reduction (○) suggested that it was in accord with further PAO oxidation of di-oxidized spermine, or possibly auto-oxidation (see Fig. 2). The reduction was inhibited by 600u/ml SOD (●) which indicated $O_2^{-\cdot}$ generation. The preparation of cytochrome contained 12·5% in its reduced state, which was first fully oxidized (0–3 h) before partial reduction (3–7 h) giving a total measurement of 20–25% reduction.

FIG. 4.—Representative graph. Inhibition of Bri8 lymphocyte proliferation by interaction of spermine with PAO in foetal calf serum (○) and its potentiation by 300 u/ml SOD (Batch 38C-8190) (●). Addition of 100 u/ml SOD gave no potentiation (not shown). Batch 7017 SOD gave only about a 2-fold potentiation (300 u/ml, not shown). Each point represents the mean of triplicate assays (mean ± s.d.). Results statistically self-evident.

Thus, rather than contribute to cell damage as suspected, destruction of any $O_2^{-\cdot}$ generated during spermine catabolism apparently enhanced cytotoxicity, perhaps by delaying the further oxidation of di-oxidized spermine. It is therefore unlikely that $O_2^{-\cdot}$, if generated in cultures, reached a level sufficient to elicit cytotoxicity directly. It is noteworthy that extremely high levels of PAO–substrate mixture were needed to show cytochrome *c* reduction, but the number of sites on the relatively large cytochrome molecule that are reducible is not taken into account.

The SOD preparations were not contaminated by PAO as shown by the radiochemical assay. Nonetheless it is doubtful whether the different batches were comparable in activity or purity (Wardman, 1979).

Thiourea, which is a well-known ·OH scavenger, reproducibly afforded significant protection of cells against the cytostasis due to spermine oxidation (50% protection at 1·0 mM). In marked contrast, other at least equally powerful ·OH scavengers afforded no discernible protection at maximal concentrations tolerated for cell cultures (PTTU, 0·2 mM; DMSO, 2·0% v/v; benzoate, 2·0 mM; butanol, 0·1% v/v; ethanol, 0·75% v/v). Thiourea apparently produced this reversal by competing in great excess with natural substrate for PAO, since it also suppressed the chemiluminescence of the PAO–substrate interaction (see below).

*Inhibitors of anion permeability*

SITS and ANS suppress exchange of anions, including $O_2^{-\cdot}$, across plasma membranes of those cells which possess anion

FIG. 5.—Chemiluminescence following interaction of 300$\mu$M spermine with 1·0 mg/10·0 ml purified PAO in the presence of 100$\mu$M luminol in PBS (pH 7·2 at 28°C) (A). Luminescence was equal when the Luminal was added 13·0 min after mixing reagents (arrowed) showing that light emission was not attributable to short-lived $O_2$-dependent free radicals. Autoluminescence of reaction mixture in absence of luminol (B). Autoluminescence of reaction mixture in the absence of PAO (C).

FIG. 6.—Inhibition of PAO-substrate chemiluminescence by catalase at 55·0 u/ml (D), 110·0 u/ml (E) and 1,100 u/ml (F). Reagent concentrations as in Fig. 5. Curve (A) from Fig. 5.

FIG. 7.—Inhibition of PAO-substrate chemiluminescence by thiourea at 0·05 mM (H), 0·01 mM (I), 0·2 mM (J) and 2·0 mM (K), and insignificant inhibition by PTTU (0·1mM) at maximal tolerated level for cell culture (L). Reagent concentrations as in Fig. 5. Curve (A) from Fig. 5.

channels (*vide* Gennaro & Romeo, 1979). When added to cultured lymphocytes at maximal tolerated concentrations of 500 $\mu$M SITS and 5·0$\mu$M ANS, they did not modulate cytotoxicity elicited by spermine oxidation. Thus anions either did not participate directly in the cytotoxicity or (less likely), anion channels do not exist in Bri8 lymphocytes.

### *Chemiluminescence*

PAO–substrate interaction produced much chemiluminescence in the presence of luminol (Fig. 5). Oxidation of luminol produces light emission (Weber *et al.*, 1943). Two agents could be formed which should oxidize luminol: $OH^-$ (*e.g.* $NH_3 + H_2O \rightleftharpoons NH^+_4 + OH^-$) and $H_2O_2$. The luminescence was ablated by added catalase (Fig. 7) and so was attributed to $H_2O_2$ byproduct and not free radicals. The luminescence was bi-phasic. Whereas the initial phase of luminescence was in accord with the process of spermine oxidation as judged by a radiochemical assay, despite extensive investigation the cause of the final phase was not ascertained. Enzyme activity was indicated by the height of the primary luminescence peak (inverse relationship). The chemiluminescence was inhibited by thiourea (Fig. 6) but not by PTTU, DMSO, benzoate or alcohols, at concentrations tolerated for lymphocyte culture. Chemiluminescence was also inhibited by 3-hydroxybenzyloxyamine ($ID_{50} < 1{\cdot}0$ $\mu$M), semicarbazide ($ID_{50} = 50{\cdot}0$ $\mu$M) and aminoguanidine ($ID_{50} < 250$ $\mu$M). Relatively low but nonetheless measurable chemiluminescence was produced by PAO–substrate interaction in the absence of luminol (Fig. 5).

Though requiring some clarification, the technique is remarkably accurate and simple and could be adapted for assay of both PAO and substrate in tissues.

### DISCUSSION

In a recent publication (Gaugas & Dewey, 1979) we have discussed the phenomenon of cytostasis of mammalian cells resulting from the *in vitro* enzymic oxidation of spermine. Evidence has now been presented that $O_2$-dependent free radicals were not apparently direct participants in the system. The findings generally support circumstantial evidence that labile di-oxidized spermine was the agent responsible for $G_1$ arrest of cell proliferation (Gaugas & Dewey, 1979). Nonetheless, it is feasible that the free radicals could be generated during the catabolism of di-oxidized spermine and provide an additional antimitotic agent. As we shall argue later, free-radical participation might accord with modifying intracellular events. Moreover, participation of free radicals on largely theoretical grounds cannot be ignored, and is therefore discussed. Enzymically produced radicals need not cause cell damage, because they may fail to detach from the enzyme and diffuse to a "wrong" substrate in order to react cytotoxically (Yamazaki, 1977); they are scavenged by thiols, or enzymes such as SOD, catalase and endogenous glutathione peroxidase (Pryor, 1978).

As a precautionary note, when thiourea was added to cultured lymphocytes because it is a well known scavenger of $^{\cdot}OH$, the results conflicted with those obtained using other known scavengers. Thiourea was alone in affording protection against the cytostasis system, yet undoubtedly reacted in some capacity other than as an $^{\cdot}OH$ scavenger.

If $O_2^{-\cdot}$ was generated in a foetal calf serum (PAO) and spermine mixture in lymphocyte cultures, it did not arise until a further stage of oxidation of di-oxidized spermine. Such $O_2^{-\cdot}$ was not deleterious to cell viability when the concentration of the reaction mixture was optimal for cytostasis. Obviously, such cells had already been exposed to di-oxidized spermine, so evaluation of an $O_2^{-\cdot}$ effect was made by showing that the addition of SOD to destroy $O_2^{-\cdot}$ failed to reduce the cytotoxicity measured throughout a spermine dose–response curve. Thus antiproliferative ability due even in part to $O_2^{-\cdot}$ activity was not forthcoming. It seems likely that insufficient $O_2^{-\cdot}$ was generated for itself to contribute to the cytotoxicity. Serum ceruloplasmin should have influenced the results, since it scavenges $O_2^{-\cdot}$ (Goldstein *et al.*, 1979). Serum could also possibly contain traces of SOD able to destroy the relatively small amount of $O_2^{-\cdot}$, as it was slowly generated by reaction mixtures.

Curiously, it was found that SOD potentiated the cytostasis of spermine oxidation. If $O_2^{-\cdot}$ is catalytic for oxidation of di-oxidized spermine by PAO, its destruction should help stabilize this otherwise labile primary product.

It is feasible that $O_2^{-\cdot}$ reacted with a likely non-cytotoxic and relatively stable carboxyl derivative of di-oxidized spermine ($RHC{=}O + \frac{1}{2}O_2 \rightarrow RCOOH$) to generate a free radical (*i.e.* $RCOOH + O_2^{-\cdot} \rightarrow RCO^{\cdot} + HO^{-} + O_2$) which could be the cytotoxic agent (see Peters & Foote, 1976). In other words, $O_2^{-\cdot}$ might regenerate a cytotoxin. Di-oxidized spermine could also generate a free radical (RCO·) by auto-oxidation (Nonhebel & Walton, 1974).

The reaction pathway leading to cytotoxicity from $O_2^{-\cdot}$ is unknown. Of the relatively few biological pathways recognized for $O_2^{-\cdot}$ deployment (Oberley & Buettner, 1979) in respect of polyamine oxidation, the sulphydryls are of particular interest,

$$RSH + H^+ + O_2^{-\cdot} \rightarrow$$

$$RS^\cdot + H_2O_2 \xrightarrow{+RS^\cdot} RSSR$$

since they have been shown both to enhance and protect against polyamine–oxidation–elicited cytostasis, according to concentration (Dewey, unpublished). Cytotoxicity of $O_2$-dependent free radicals, due to irreversible cross-linkage of essential cell proteins at nucleophilic sulphydryl residues, has been suggested (Tse *et al.*, 1976). On the other hand, the aldehyde moiety of di-oxidized spermine could adduct with cysteine (Schauenstein *et al.*, 1977).

Diminished endogenous levels of SOD, and $O_2^{-\cdot}$ production, occur almost invariably in tumours. Diminished SOD has been associated with rapid proliferation in non-malignant cells, thus implicating its $O_2^{-\cdot}$ substrate in the regulation of cell proliferation (reviewed by Oberley & Buettner, 1979). As well as exogenous PAO in cultures, amine oxidases also occur in cells (Kapeller–Adler, 1970; Quash *et al.*, 1979; Morgan *et al.*, 1980) so a source of $O_2$-dependent free radicals could be from intracellular overall oxidation of polyamines, or indeed oxidized diamines (unpublished) or oxidized monoamines (Cohen, 1978; Borg *et al.*, 1978). This hypothesis is subject to the unlikely event of $O_2^{-\cdot}$ being not merely a product of the reaction mixture *in vitro*, but an *in vivo* physiological or pathological product. The $O_2^{-\cdot}$ is very diffusible into cells and tissues (Lynch & Fridovich, 1978) and thereby could have evaded dismutation by extracellular SOD. The addition of SITS and ANS, which suppress anion but not cation permeability of cells (*vide* Gennaro & Romeo, 1979) failed to alter the susceptibility of lymphocytes to polyamine-oxidation cytostasis. Though anion channels are present in granulocytes, their existence in lymphocytes has not been ascertained (Gennaro & Romeo, 1979).

As well as being a byproduct of enzymic spermine oxidation, additional $H_2O_2$ could be formed from both PAO oxidation of di-oxidized spermine and from $O_2^{-\cdot}$ (McCord & Fridovich, 1969).

$$O_2^{-\cdot} + O_2^{-\cdot} + 2H^+ \xrightarrow{SOD} H_2O_2 + O_2$$

Incidentally, this raises the important consideration of whether cells are either more or less vulnerable to exogenous rather than endogenous $H_2O_2$, or indeed free radicals. In the system, the participation of $H_2O_2$ from oxidation of spermine was readily excluded because of its destruction by added catalase. In contrast, the intracellularly generated $H_2O_2$, if any, could not be similarly excluded. However, the cell should be protected against any $H_2O_2$ toxicity by cytosol peroxidases (Salin & McCord, 1974).

Unlike aldehyde compounds in general, which are indiscriminately toxic throughout the cell cycle, N,N′-*bis*(3-propionaldehyde)-1,4 butanediamine (di-oxidized spermine) apparently caused $G_1$ arrest (Gaugas & Dewey, 1979). Intracellular conversion of di-oxidized spermine itself to a free-radical state (*e.g.* RCO·) or generation of $O_2^{-\cdot}$, $HO_2^{-\cdot}$ or ·OH and $H_2O_2$, could be mandatory for cytotoxic activity. If $O_2^{-\cdot}$ were generated, cells with diminished SOD (*i.e.* tumour cells) should then be more susceptible to cytostasis. Our results suggest, however, that such cells would be less susceptible to the cytotoxic activity of di-oxidized spermine. The products of biochemical events accompanying extracellular spermine catabolism would then be modified intracellularly, especially since PAO, SOD and catalase (but not the products) would be excluded by molecular size from cell entry. The restricted location of endogenous macromolecular PAO, SOD and peroxidase, in plasma membrane, organelles or cytosol could determine the fate and any interrelationship function of di-oxidized spermine and byproducts once incorporated into a cell. Such states should alter in different phases of the cell cycle, possibly in accordance with the $G_1$ arrest elicited by exogenous spermine oxidation. The system could integrate into permutations of biochemical pathways to arrest cell proliferation, or to protect against arrest. For an example so far not mentioned, RHC = O could be converted to innocuous carboxyl (RCOOH) by endogenous aldehyde reductase or xanthine oxidase but with $O_2^{-\cdot}$ generation.

Though many polyamine-unrelated aldehyde compounds are involved as intermediates in normal cell metabolism, they are not toxic (Schauenstein *et al.*, 1977). Rapid detoxification of di-oxidized spermine in the cells is necessary if catabolism of polyamines is a function of mitochondrial PAO. Thus it seems unlikely that the relatively slow further oxidation of dioxidized spermine and therefore $O_2^{-\cdot}$ generation would occur *in vivo*. Moreover, because the carbonyl products of further oxidation (Kimes & Morris, 1971) should themselves be cytotoxic (Alarcon, 1970) the total process would give no advantage to the cell. Dioxidized spermine itself might reduce a vital cell component essential for $G_1$ metabolism.

In conclusion, $O_2$-dependent free radicals or $H_2O_2$ generated by polyamine catabolism appear not to be involved in *in vitro* exogenous cytostasis. Their production intracellularly from oxidized polyamine is, however, feasible. It was important to determine any role for direct participation of free radicals, since the purported antiproliferative potency of dioxidized spermine is of interest in developmental chemotherapeutics for malignancy. The synthesized and chemically stable ethyl-acetal derivatives of oxidized polyamines are thought to owe their latent potent *in vivo* antileukaemic effects to hydrolysis which generates an aldehyde moiety (Allen *et al.*, 1979). On the other hand, a slow secondary oxidation by PAO may occur, to generate toxic levels of $O_2^-$ and/or carbonyls. If so, our findings suggest that on an equimolar basis the ethyl-acetal derivatives would be less antiproliferative than di-oxidized spermine itself.

We thank the Cancer Research Campaign for support, and Miss A. R. Galpine for discussion.

### REFERENCES

ALARCON, R. A. (1970) Acrolein: IV. Evidence for the formation of the cytotoxic aldehyde acrolein from enzymatically oxidized spermine and spermidine. *Arch. Biochem. Biophys.*, **137**, 365.

ALLEN, J. C., SMITH, C. J. & HUSSAIN, J. I. (1979) The antimitotic effect of synthetic derivatives of oxidized polyamines on lymphocytes. *Cell Tissue Kinet.*, **13** 183.

BACHRACH, U. (1973) *Function of Naturally Occurring Polyamines*. New York: Academic Press. p. 82.

BACHRACH, U. & PERSKY, S. (1964) Antibacterial activity of oxidized spermine. *J. Gen. Microbiol.*, **37**, 195.

BORG, D. C., SCHAICH, K. M., ELMORE, J. J., JR & BELL, J. A. (1978) Cytotoxic reaction of free radical species of oxygen. *Photochem. Photobiol.*, **28**, 887.

BYRD, W. J., JACOBS, D. M. & AMOSS, M. S. (1977) Synthetic polyamines added to cultures containing bovine sera reversibly inhibit *in vitro* parameters of immunity. *Nature*, **267**, 621.

COHEN, G. (1978) The generation of hydroxyl radicals in biologic systems: Toxological aspects. *Photochem. Photobiol.*, **28**, 669.

FEE, J. A. & VALENTINE, J. S. (1977) Chemical and physical properties of superoxide. In *Superoxide and Superoxide Dismutase*. Ed. Michelson, McCord & Fridovich. New York: Academic Press. p. 30.

FULLER, D. J. M., GERVER, E. W. & RUSSELL, D. H. (1977) Polyamine biosynthesis and accumulation during $G_1$-phase of transition. *Cell Physiol.*, **93**, 81.

GAUGAS, J. M. & CURZEN, P. (1978) Polyamine interaction with pregnancy serum in suppression of lymphocyte transformation. *Lancet*, i, 18.

GAUGAS, J. M. & DEWEY, D. L. (1979) Evidence for serum binding of oxidized spermine and its potent $G_1$-phase inhibition of cell proliferation. *Br. J. Cancer*, **39**, 548.

GENNARO, R. & ROMEO, D. (1979) The release of superoxide anion from granulocytes: Effect of inhibitors of anion permeability. *Biochem. Biophy Res. Commun.*, **88**, 44.

GOLDSTEIN, I. M., KAPLAN, H. B., EDELSON, H. S. & WEISSMANN, G. (1979) Ceruloplasmin. A scavenger of $O_2^{-\cdot}$ anion radicals. *J. Biochem.*, **254**, 4040.

HABER, F. & WEISS, J. (1934) see FERRANDINI, C., FOOS, J., HOUEE, C. & PUCHEAULT, J. (1978) The reaction between superoxide anion and hydrogen peroxide. *Photochem. Photobiol.*, **28**, 697.

Höltta, E. (1977) Oxidation of spermidine and spermine in rat liver: Purification and properties of polyamine oxidase. *Biochemistry*, **16**, 91.

Kapeller-Adler, R. (1970) *Amine Oxidases and Methods for Their Study*. New York: Wiley. p. 1.

Kimes, B. W. & Morris, D. R. (1971) Preparation and stability of oxidized polyamines. *Biochim. Biophys. Acta*, **228**, 223.

Knoll, J. (1976) Analysis of the pharmacological effects of selective monoamine oxidase inhibitors. In *Monoamine Oxidase and Its Inhibition*. Ciba Found. Symp. **39** (new series). Amsterdam: Elsevier. p. 135.

Lynch, R. E. & Fridovich, I. (1978) Effects of superoxide on the erythrocyte membrane. *J. Biol. Chem.*, **253**, 1838.

McCord, J. M. & Fridovich, I. J. (1969) Superoxide dismutase. An enzymic function for erythrocuprein (hemocuprein). *J. Biol. Chem.*, **244**, 6049.

McCord, J. M. (1974) Free radicals and inflammation; Protection of synovial fluid by superoxide dismutase. *Science*, **185**, 529.

Melvin, M. A. L. & Keir, H. M. (1978) Diminished excretion of polyamines from BKH-21/C13 cells exposed to methylglyoxal *bis*(guanylhydrazone). *Biochem. J.*, **174**, 349.

Morgan, D. M. L., Ferulga, J. & Allison, A. C. (1980) Polyamine oxidase and macrophage function. In *Polyamines in Biomedical Research*. Ed. Gaugas. New York: Wiley.

Myers, L. S., Jr (1973) Free radical damage of nucleic acids and their components by ionizing radiation. *Fed. Proc.*, **32**, 1882.

Newton, N. E. & Abdel-Monem, M. M. (1978) Inhibitors of polyamine biosynthesis. 4. Effects of α-methyl-(±)-ornithine and methylglyoxal *bis*-(guanylhydrazone) on growth and polyamine content of L1210 leukaemic cells of mice. *J. Med. Chem.*, **20**, 249.

Nonhebel, D. C. & Walton, J. C. (1974) Autooxidation. In *Free-radical Chemistry*. Cambridge: University Press. p. 393.

Oberley, L. W. & Buettner, G. R. (1979) Role of superoxide dismutase in cancer: A review. *Cancer Res.*, **39**, 1141.

Peters, J. W. & Foote, C. S. (1976) Roll of superoxide dismutase in cancer: A review. *Cancer Res.*, **39**, 1141.

Pryor, W. A. (1978) The formation of free radicals and the consequences of their reactions *in vivo*. *Photochem. Photobiol.*, **28**, 787.

Quash, G., Keolouangkhot, T., Gazzolo, L., Ripoli, H. & Saez, S. (1979) Diamine oxidase and polyamine oxidase activities in normal and transformed cells. *Biochem. J.*, **177**, 275.

Rijke, E. O. & Ballieux, R. E. (1978) Is thymus-derived lymphocyte inhibitor a polyamine? *Nature*, **274**, 804.

Sagone, A. L., Jr, Kamps, S. & Campbell, R. (1978) The effect of oxidant injury on the lymphoblastic transformation of human lymphocytes. *Photochem. Photobiol.*, **28**, 909.

Salin, M. L. & McCord, J. M. (1974) Superoxide dismutase in polymorphonuclear leukocytes. *J. Clin. Invest.*, **54**, 1005.

Schauenstein, E., Esterbauer, H. & Zollner, H. (1977) *Aldehydes in Biological Systems*. Transl. by P. Gore. London: Pion. pp. 4 & 163.

Tabor, C. W., Tabor, H. & Bachrach, U. (1964) Identification of the aldehydes produced by the oxidation of spermine with purified plasma amine oxidase. *J. Biol. Chem.*, **239**, 2194.

Tabor, C. W. & Tabor, H. (1976) 1,4-diaminobutane (putrescine), spermidine and spermine. *Ann. Rev. Biochem.*, p. 285.

Tse, D. C. S., McCreery, R. L. & Adams, R. N. (1976) Potential oxidation pathways of brain catecholamines. *J. Med. Chem.*, **19**, 37.

Wardman, P. (1979) Specificity of superoxide dismutase in catalysing redox reactions: A pulse radiolysis study. In *Radiation Biology and Chemistry: Research Developments*. Eds Edwards *et al.* Amsterdam: Elsevier. p. 91.

Weber, K., Lahn, W. & Hieber, E. (1943) Über die luminescenz des luminols, IV. Mitteil: Die chemiluminescenz des luminols bei anwesenheit von rutheniumchlorid und vanadylsulfat. *Ber.*, **76**, 366.

Yamazaki, I. (1977) *Free Radicals in Biology*. Vol. III. Ed. Pryor. New York: Academic Press. p. 183.